# Creep damage model of rock considering the influence of fractional order and temperature

**DOI:** 10.1371/journal.pone.0351537

**Published:** 2026-07-08

**Authors:** Yuemei Li

**Affiliations:** School of Civil Engineering, Liaodong University, Dandong, China; Faculty of Engineering, University of Rijeka, CROATIA

## Abstract

To address the shortcomings of traditional rock creep models, including neglecting the time-varying cumulative effect of thermal damage, low accuracy in describing accelerated creep, and failing to reflect the thermo-mechanical coupling rheological mechanism of deep high-temperature rock mass, this paper proposes a fractional-order rock creep damage model considering temperature influence, based on continuum damage mechanics and fractional calculus theory. Firstly, a time-varying thermal damage evolution equation dependent on both temperature level and heating duration is developed, and a temperature-coupled stress creep damage equation is established synchronously to realize the synergistic evolution of dual time-varying damage, with damage only acting on the fractional viscosity coefficient to keep the elastic modulus unchanged. Then, combined with the improved fractional-order Kelvin element and optimized Nishihara model, the uniaxial creep damage model is derived via rigorous equation derivation and integration, and the corresponding triaxial model is further deduced for complex engineering stress conditions. Finally, model verification and comparison are conducted based on rock creep tests under 25 °C, 50 °C, 75 °C and 100 °C. The results indicate that the model can accurately characterize the full three-stage creep behavior of rock, with the predicted curves in excellent agreement with test data and all correlation coefficients exceeding 0.95. Compared with the classical Nishihara model and existing models, the proposed model has higher fitting precision and better performance in capturing accelerated creep inflection points, verifying its superiority and reliability, and providing theoretical support for long-term stability analysis of deep high-temperature rock engineering.

## 1 Introduction

With the continuous expansion of the construction scale of deep mineral resources exploitation, geothermal energy development, deep disposal of nuclear waste, deep tunnel and chamber engineering, the deep occurrence environment of rock engineering is becoming more and more complex, which is typical of the multiple coupling effect of high ground temperature, high stress and long time load [1–3]. Under the combined action of high temperature aging and constant load for a long time, the mechanical behavior of deep rock mass shows significant creep characteristics, and the temperature will not only directly change the viscoelastic rheological properties of rock, but also cause irreversible thermal degradation effects such as mineral phase transformation, grain boundary cracking and pore expansion. With the initiation, expansion and penetration of microcracks under stress, the synergistic evolution law of thermal damage and stress creep damage is formed, which ultimately controls the long-term deformation and failure of rock and the stability of engineering structure [4–6]. Therefore, it is of great theoretical value and engineering practical significance for the long-term stability evaluation, structural design and safety warning of deep rock engineering to construct a rock creep damage model that can accurately reflect the temperature-time-stress coupling effect and take into account the double time-varying evolution law of thermal damage and stress damage [7].

Scholars at home and abroad have carried out a lot of research on rock creep characteristics [8]. The traditional integer-order creep model (such as Nishihara model, Burgess model, generalized Kelvin model, etc.) is based on the classical viscoelastic theory, which can preliminarily describe the deformation law of rock deceleration creep and steady-state creep stage. However, it uses integer-order derivative to characterize the rheological properties, which is difficult to accurately fit the nonlinear and unsteady viscoelastic rheological behavior of rock, and the characterization accuracy of accelerated creep stage is insufficient [9,10]. The introduction of fractional calculus theory effectively makes up for this defect. Through the continuous adjustment of fractional order, the memory and genetic rheological properties of rock can be accurately described by fewer parameters, which has become the mainstream direction of rock nonlinear creep research [11].

The four relevant literatures have carried out targeted research on rock creep characteristics and constitutive models, laying a solid foundation for this study. Wang et al. [12] focuses on fractured rock, establishes a creep constitutive model considering nonlinear creep degradation, and optimizes the description of creep deformation driven by internal fracture damage. Lyu et al. [13] carries out ultra-long-term creep tests on salt rock, and proposes a nonlinear creep-damage constitutive model to reveal the long-term rheological law of salt rock for underground storage engineering. Liu et al. [14] constructs a rock creep model based on the time-dependent degradation characteristics of rock strength, linking strength attenuation with creep damage evolution. Qiao et al. [15] develops a rock creep constitutive model based on internal state variables, characterizing the internal structural damage and full-stage creep behavior of rock via continuum mechanics theory.

The existing fractional-order creep models mostly focus on the influence of stress single factor. Li & Jia [16] proposed a fractional-order nonlinear creep damage model, while Yang et al. [17] developed a fractional creep model incorporating multiscale damage to better describe accelerated creep. However, existing studies rarely consider the time-varying cumulative effect of thermal damage and the synergistic coupling mechanism of temperature-stress dual damage, failing to fully reflect the thermo-mechanical coupling rheological behavior of deep high-temperature rock masses. Some models introduce the correction of temperature to the viscosity coefficient, only consider the influence of temperature on the rheological rate of rock, and ignore the cumulative evolution effect of thermal damage with time under the long-term effect of high temperature. It is difficult to reflect the continuous deterioration of rock mechanical properties with the duration of high temperature in deep high temperature environment.

In view of the shortcomings of the existing research, based on the continuum damage mechanics and fractional calculus theory, this paper fully considers the actual mechanical mechanism of high temperature creep of deep rock, and focuses on breaking through the limitation of thermal damage as a static variable in the traditional model. The time-varying thermal damage evolution equation that depends on both temperature level and high temperature action time is constructed, and the stress creep damage equation coupled with temperature effect is established synchronously to realize the synergistic coupling of double time-varying damage. The temperature effect is introduced into the fractional-order dashpot element to correct the temperature dependence of the fractional-order rheological parameters. Combined with the improved fractional-order viscoelastic-viscoplastic creep framework, a full-stage rock creep damage model considering time-varying thermal damage, temperature-dependent fractional-order rheology and double damage coupling is derived. Through rigorous differential equation derivation and integral solution, the explicit analytical expression of the model is given, and the physical meaning and calibration method of each parameter of the model are clarified. Finally, the rationality and applicability of the model are verified by comparing with the relevant experimental data. The model in this paper can accurately characterize the full-stage deformation law of rock deceleration creep, steady-state creep and accelerated creep, and comprehensively reflect the co-evolution mechanism of thermal damage and stress damage under long-term high temperature, which can provide theoretical support for long-term stability analysis and rheological disaster prevention and control of deep high-temperature rock engineering.

## 2 Temperature damage variable and stress damage variable

According to the scalar damage theory of continuum damage mechanics (CDM), the damage variable D∈[0,1): *D* = 0 represents no damage to the rock. D→1 represents the complete failure of rock. Mineral dehydration, phase transformation, grain boundary slip, thermal cracking, and pore structure deterioration occur in rocks at high temperatures. These deteriorations depend on both the temperature level *T* and the high temperature action time *t*. Therefore, the thermal damage must be $D_{T}(T,t)$. The thermal damage rate is defined to satisfy *t*he Weibull-type nonlinear evolution. The evolution rate of thermal damage variable can be expressed as


$$\begin{array}{c} \frac{dD_{T}}{dt}=\frac{p}{t_{c}}{\left(\frac{t}{t_{c}}\right)}^{p-1}\exp\left[-{\left(\frac{T-T_{0}}{T_{c}}\right)}^{n}{\left(\frac{t}{t_{c}}\right)}^{p}\right] \end{array}$$ (1)

where *p* is the time shape parameter, $t_{c}$ is the thermal damage characteristic time, *n* is the temperature shape parameter, $T_{c}$ is the thermal damage characteristic temperature, and *T*_0_ is the reference temperature without obvious thermal damage, *t* is the creep time.

The initial condition is


$$\begin{array}{c} t=0,\;D_{T}(0)=0 \end{array}$$ (2)

By integrating Eq. (1) with Eq. (2), we get


$$\begin{array}{c} D_{T}=1-\exp\left[-{\left(\frac{T-T_{0}}{T_{c}}\right)}^{n}{\left(\frac{t}{t_{c}}\right)}^{p}\right] \end{array}$$ (3)

When the stress exceeds the long-term strength $\sigma_{s}$, the microcracks expand and penetrate. The increase of temperature reduces the activation energy and accelerates the damage. At this time, the damage rate is positively correlated with the effective stress, temperature and time. The Kachanov-Rabotnov type damage rate is adopted and Arrhenius temperature effect is introduced. At this time, the newly increased stress damage variable evolution rate $D_{\sigma }(t)$ is


$$\begin{array}{c} \frac{dD_{\sigma }}{dt}=\omega (T)<\sigma -\sigma_{s}>\left(1-D_{\sigma }\right) \end{array}$$ (4)

where ω(T) is the temperature dependent damage rate factor. $<\sigma -\sigma_{s}>$ is the switching function, $\sigma_{s}$ is the long-term strength, σ is the stress.

The temperature dependent damage rate factor ω(T) can be expressed as


$$\begin{array}{c} \omega (T)=\omega_{0}\exp\left(-\frac{Q_{\omega }}{RT}\right) \end{array}$$ (5)

where $Q_{\omega }$ is the damage activation energy, *R* is the gas constant, and *T* is the absolute temperature, $\omega_{0}$ is the initial temperature dependent damage rate factor.

The switching function $<\sigma -\sigma_{s}>$ can be expressed as


$$\begin{array}{c} <\sigma -\sigma_{s}>=\left\{ \begin{array}{cc} 0 & \sigma <\sigma_{s}\\ \sigma -\sigma_{s} & \sigma \geq \sigma_{s} \end{array}\right. \end{array}$$ (6)

The initial condition is


$$\begin{array}{c} t=0,\;D_{\sigma }(0)=0 \end{array}$$ (7)

By integrating Eq. (4) with Eq. (7), we get


$$\begin{array}{c} D_{\sigma }=1-\exp\left[-\omega_{0}\exp\left(-\frac{Q_{\omega }}{RT}\right)<\sigma -\sigma_{s}>t\right] \end{array}$$ (8)

The total damage variable *D* can be expressed as


$$\begin{array}{c} D=1-\left(1-D_{T}\right)\left(1-D_{\sigma }\right) \end{array}$$ (9)

By substituting Eqs. (3) and (8) into Eq. (9), we obtain

When $\sigma \leq \sigma_{s}$


$$\begin{array}{c} D=1-\exp\left[-{\left(\frac{T-T_{0}}{T_{c}}\right)}^{n}{\left(\frac{t}{t_{c}}\right)}^{p}\right]=1-\exp\left[-Z_{T}{\left(\frac{t}{t_{c}}\right)}^{p}\right] \end{array}$$ (10)

When $\sigma >\sigma_{s}$,


$$\begin{array}{c} D=1-\exp\left[-{\left(\frac{T-T_{0}}{T_{c}}\right)}^{n}{\left(\frac{t}{t_{c}}\right)}^{p}\right]\exp\left[-\omega_{0}\exp\left(-\frac{Q_{\omega }}{RT}\right)<\sigma -\sigma_{s}>t\right] \end{array}$$ (11)

where $Z_{T}$ and $Z_{\sigma }$ are replacement parameter.

## 3 Creep model considering the effects of temperature and stress

### 3.1 Uniaxial creep model

The traditional Nishihara model cannot well describe the accelerated creep deformation law of rock, nor can it reflect the deformation characteristics of rock during high temperature. Therefore, the traditional Nishihara model needs to be improved. The instantaneous strain $\varepsilon_{1}$ can be expressed as [18]


$$\begin{array}{c} \varepsilon_{1}=\frac{\sigma }{E_{1}} \end{array}$$ (12)

where σ is the stress, *E*_1_ is the instantaneous elastic modulus.

In the viscoelastic strain stage, the rock is only affected by time and temperature, and the influence of stress on it is less than the above two factors. In this paper, fractional derivative is used. Its expression is [19]


$$\begin{array}{c} \frac{du}{dt^{\beta }}=\lim_{t^{\prime }\to t}\frac{u(t)-u\left(t^{\prime }\right)}{t^{\beta }-t^{\prime \beta }}=\frac{1}{\beta t^{\beta -1}}\frac{du}{dt} \end{array}$$ (13)

where β is the time fractal dimension, *u*(*t*) is a specific function, $u^{\prime }(t)$ is a specific function derivative.

In the context of rock creep, the fractional order β defined as the time fractal dimension essentially quantifies the material’s memory-dependent and nonlinear rheological behavior: it bridges the gap between purely elastic (β=0) and perfectly viscous (β=1) responses, with values between 0 and 1 reflecting the combined elastic and viscous contributions to deformation, where a smaller β indicates stronger elastic memory and faster creep decay, while a larger β closer to 1 denotes dominant viscosity and more pronounced nonlinear flow. This parameter also evolves with temperature and stress-induced damage, as thermal degradation and microcrack propagation weaken the rock’s elastic skeleton and enhance viscous behavior, increasing β and accelerating the transition to accelerated creep. It thus provides a physically meaningful, self-consistent measure of the material’s time-dependent structural evolution, rather than being merely a mathematical fitting coefficient.

The proposed fractional-order Hausdorff derivative is a local operator defined on a fractal time metric, yet it can effectively capture the non-local memory and time-dependent behavior of rock creep by embedding the “memory effect” into the scaling structure of the time itself. Unlike standard integer-order derivatives, which describe only the instantaneous rate of change, this derivative introduces a time-scaling factor $t^{\beta -1}$ through the fractal time dimension β. This scaling factor acts as a time-dependent weight function, automatically assigning stronger influence to the recent past and weaker influence to the distant past, which mimics the fading memory of creep deformation. The parameter β directly controls the strength of this memory. As β deviates from 1, the time metric becomes increasingly non-linear, making the derivative sensitive to the entire deformation history rather than just the current moment. Consequently, even though the operator is defined locally, its fractal scaling law allows it to reproduce the characteristic three-stage creep behavior (primary, steady-stable, and accelerating) and the history-dependent rheological properties of rock, thus serving as a mathematically simpler yet physically meaningful alternative to non-local fractional operators.

The fractional viscosity coefficient damaged $\eta_{2}(T,t)$ by temperature is defined as


$$\begin{array}{c} \eta_{2}(T,t)=\eta_{20}\left(1-D_{2}\right) \end{array}$$ (14)

where $\eta_{20}$ is the initial viscosity coefficient damaged by temperature, *D*_2_ is the viscoelastic damage variable.

By substituting Eq. (10) into Eq. (14), we obtain


$$\begin{array}{c} \eta_{2}(T,t)=\eta_{20}\exp\left[-Z_{2T}{\left(\frac{t}{t_{c}}\right)}^{p_{2}}\right] \end{array}$$ (15)

where *p*_2_ is the viscoelastic time shape parameter, *Z*_2 *T*_ is viscoelastic replacement parameter.


$$\begin{array}{c} \sigma =E_{2}\varepsilon_{2}+\eta_{2}(T,t)\frac{1}{\beta_{2}t^{\beta_{2}-1}}\frac{d\varepsilon_{2}}{dt} \end{array}$$ (16)

where *E*_2_ is the viscoelastic elastic modulus, $\varepsilon_{2}$ is the viscoelastic strain, $\varepsilon_{2}^{\prime }$ is the viscoelastic strain rate, $\beta_{2}$ is the viscoelastic time fractal dimension.

The fractional-order Kelvin creep model considering temperature damage variable is expressed as By substituting Eq. (15) into Eq. (16), we obtain


$$\begin{array}{c} \sigma =E_{2}\varepsilon_{2}+\eta_{20}\exp\left[-Z_{2T}{\left(\frac{t}{t_{c}}\right)}^{p_{2}}\right]\frac{1}{\beta_{2}t^{\beta_{2}-1}}\frac{d\varepsilon_{2}}{dt} \end{array}$$ (17)

The initial condition is


$$\begin{array}{c} t=0,\;\varepsilon_{2}(0)=0 \end{array}$$ (18)

Eq. (17) is arranged in the form of strain rate.


$$\begin{array}{c} \frac{\eta_{20}}{\sigma -E_{2}\varepsilon_{2}}d\varepsilon_{2}=\beta_{2}t^{\beta_{2}-1}\exp\left[Z_{2T}{\left(\frac{t}{t_{c}}\right)}^{p_{2}}\right]dt \end{array}$$ (19)

By integrating both sides of Eq. (19) from 0 to *t*, we obtain


$$\begin{array}{c} \int_{0}^{\varepsilon_{2}}\frac{\eta_{20}}{\sigma -E_{2}\varepsilon_{2}}d\varepsilon_{2}=\int_{0}^{t}\beta_{2}\tau^{\beta_{2}-1}\exp\left[Z_{2T}{\left(\frac{\tau }{t_{c}}\right)}^{p_{2}}\right]d\tau \end{array}$$ (20)

The integral of Eq. (20) is the standard form of incomplete gamma function.


$$\begin{array}{c} \int_{0}^{t}\beta_{2}\tau^{\beta_{2}-1}\exp\left[Z_{2T}{\left(\frac{\tau }{t_{c}}\right)}^{p_{2}}\right]d\tau =\frac{\beta_{2}t^{\beta_{2}}}{p_{2}}{\left[-Z_{2T}{\left(\frac{t}{t_{c}}\right)}^{p_{2}}\right]}^{-\frac{\beta_{2}}{p_{2}}}\Gamma \left[\frac{\beta_{2}}{p_{2}},-Z_{2T}{\left(\frac{t}{t_{c}}\right)}^{p_{2}}\right] \end{array}$$ (21)

By substituting the integral results into Eq. (21), we get


$$\begin{array}{c} \varepsilon_{2}=\frac{\sigma }{E_{2}}\left\{1-\exp\left\{-\frac{\beta_{2}t^{\beta_{2}}}{p_{2}}{\left[-Z_{2T}{\left(\frac{t}{t_{c}}\right)}^{p_{2}}\right]}^{-\frac{\beta_{2}}{p_{2}}}\Gamma \left[\frac{\beta_{2}}{p_{2}},-Z_{2T}{\left(\frac{t}{t_{c}}\right)}^{p_{2}}\right]\right\}\right\} \end{array}$$ (22)

For the viscoplastic model in Nishihara model, the expression after damage is

When $\sigma \leq \sigma_{s}$,


$$\begin{array}{c} \varepsilon_{3}=0 \end{array}$$ (23)

When $\sigma >\sigma_{s}$,


$$\begin{array}{c} \sigma =\eta_{3}\left(1-D_{3}\right)\frac{1}{\beta_{3}t^{\beta_{3}-1}}\frac{d\varepsilon_{3}}{dt} \end{array}$$ (24)

where $\varepsilon_{3}$ is the viscoplastic strain, $\varepsilon_{3}^{\prime }$ is the viscoplastic strain rate, $\beta_{3}$ is the viscoplastic time fractal dimension, $\eta_{3}$ is the viscoplastic fractional viscosity coefficient damaged, *D*_3_ is the viscoplastic damage variable.

By substituting Eq. (11) into Eq. (24), we obtain


$$\begin{array}{c} \sigma =\eta_{3}\exp\left[Z_{3\sigma }\left(\sigma -\sigma_{s}\right)t-Z_{3T}{\left(\frac{t}{t_{c}}\right)}^{p_{3}}\right]\frac{1}{\beta_{3}t^{\beta_{3}-1}}\frac{d\varepsilon_{3}}{dt} \end{array}$$ (25)

where *Z*_3 *T*_ and $Z_{3\sigma }$ are viscoplastic replacement parameter.

The initial condition is


$$\begin{array}{c} t=0,\;\varepsilon_{3}(0)=0 \end{array}$$ (26)

In order to make the Eq. (25) have an analytical solution, it is simplified.


$$\begin{array}{c} \sigma =\eta_{3}\exp\left[Z_{3\sigma }\left(\sigma -\sigma_{s}\right)t-Z_{3T}\left(\frac{t}{t_{c}}\right)\right]\frac{1}{\beta_{3}t^{\beta_{3}-1}}\frac{d\varepsilon_{3}}{dt} \end{array}$$ (27)

Eq. (27) is arranged in the form of strain rate.


$$\begin{array}{c} \frac{d\varepsilon_{3}}{dt}=\frac{\sigma \beta_{3}t^{\beta_{3}-1}}{\eta_{3}}\exp\left\{-\left[Z_{3\sigma }\left(\sigma -\sigma_{s}\right)t-Z_{3T}\left(\frac{t}{t_{c}}\right)\right]\right\} \end{array}$$ (28)

By integrating both sides of Eq. (28) from 0 to *t*, we obtain


$$\begin{array}{c} \int_{0}^{\varepsilon_{3}}d\varepsilon_{3}=\int_{0}^{t}\frac{\sigma \beta_{3}\tau^{\beta_{3}-1}}{\eta_{3}}\exp\left\{-\left[Z_{3\sigma }\left(\sigma -\sigma_{s}\right)\tau -Z_{3T}\left(\frac{\tau }{t_{c}}\right)\right]\right\}d\tau \end{array}$$ (29)

Therefore, the integral becomes


$$\begin{array}{c} \varepsilon_{3}=\int_{0}^{t}\frac{\sigma \beta_{3}\tau^{\beta_{3}-1}}{\eta_{3}}\exp\left\{-\left[Z_{3\sigma }\left(\sigma -\sigma_{s}\right)\tau -Z_{3T}\left(\frac{\tau }{t_{c}}\right)\right]\right\}d\tau \end{array}$$ (30)

The integral of Eq. (30) is the standard form of incomplete gamma function.


$$\begin{array}{c} \int_{0}^{t}\tau^{\beta_{3}-1}\exp\left\{-\left[Z_{3\sigma }\left(\sigma -\sigma_{s}\right)-Z_{3T}\left(\frac{\tau }{t_{c}}\right)\right]\right\}d\tau =\frac{\Gamma \left(\beta_{3}\right)-\Gamma \left(\beta_{3},Z_{3\sigma }\left(\sigma -\sigma_{s}\right)-Z_{3T}\left(\frac{t}{t_{c}}\right)\right)}{{\left[Z_{3\sigma }\left(\sigma -\sigma_{s}\right)-\frac{Z_{3T}}{t_{c}}\right]}^{\beta_{3}}} \end{array}$$ (31)

By substituting the integral results into Eq. (30), we get


$$\begin{array}{c} \varepsilon_{3}=-\frac{\sigma \beta_{3}}{\eta_{3}}{\left\{\left[Z_{3\sigma }\left(\sigma -\sigma_{s}\right)-\frac{Z_{3T}}{t_{c}}\right]t\right\}}^{-\beta_{3}}\Gamma \left\{\beta_{3},\left[Z_{3\sigma }\left(\sigma -\sigma_{s}\right)-\frac{Z_{3T}}{t_{c}}\right]t\right\} \end{array}$$ (32)

The total strain of rock ε is


$$\begin{array}{c} \varepsilon =\varepsilon_{1}+\varepsilon_{2}+\varepsilon_{3} \end{array}$$ (33)

By substituting Eqs. (12), (22), (23) and (32) into Eq. (33), we obtain

When $\sigma \leq \sigma_{s}$,


$$\begin{array}{c} \varepsilon =\frac{\sigma }{E_{1}}+\frac{\sigma }{E_{2}}\left\{1-\exp\left\{-\frac{\beta_{2}t^{\beta_{2}}}{p_{2}}{\left[-Z_{2T}{\left(\frac{t}{t_{c}}\right)}^{p_{2}}\right]}^{-\frac{\beta_{2}}{p_{2}}}\Gamma \left[\frac{\beta_{2}}{p_{2}},-Z_{2T}{\left(\frac{t}{t_{c}}\right)}^{p_{2}}\right]\right\}\right\} \end{array}$$ (34)

When $\sigma >\sigma_{s}$


$$\begin{array}{cc} \varepsilon = & \frac{\sigma }{E_{1}}+\frac{\sigma }{E_{2}}\left\{1-\exp\left\{-\frac{\beta_{2}t^{\beta_{2}}}{p_{2}}{\left[-Z_{2T}{\left(\frac{t}{t_{c}}\right)}^{p_{2}}\right]}^{-\frac{\beta_{2}}{p_{2}}}\Gamma \left[\frac{\beta_{2}}{p_{2}},-Z_{2T}{\left(\frac{t}{t_{c}}\right)}^{p_{2}}\right]\right\}\right\}\\ & -\frac{\sigma \beta_{3}}{\eta_{3}}{\left\{\left[Z_{3\sigma }\left(\sigma -\sigma_{s}\right)-\frac{Z_{3T}}{t_{c}}\right]t\right\}}^{-\beta_{3}}\Gamma \left\{\beta_{3},\left[Z_{3\sigma }\left(\sigma -\sigma_{s}\right)-\frac{Z_{3T}}{t_{c}}\right]t\right\} \end{array}$$ (35)

Eqs.(34) and (35) are the uniaxial creep damage model of rock considering the influence of fractional order and temperature.

### 3.2 Triaxial creep model

The three-dimensional derivation of rock creep damage model considering the influence of fractional order and temperature is the key to break through the one-dimensional/axisymmetric model which can only describe the simple stress state and can not reflect the real engineering stress conditions. It can not only accurately reflect the coupling effect of temperature and fractional order on rock creep memory characteristics, viscous behavior and damage evolution under the framework of three-dimensional stress tensor, but also restore the real deformation law of deep rock mass under complex thermal-mechanical environment, and meet the requirements of thermodynamic self-consistency of continuous medium. It provides a theoretical basis for the model to be implanted into numerical simulation software and serve the long-term stability analysis of underground engineering, and improves the theoretical system of rock rheological damage. It is a necessary link from indoor test law to engineering field application.

The stress tensor $\sigma_{ij}$ can be decomposed into spherical stress tensor $\sigma_{m}$ and deviatoric stress tensor $S_{ij}$. Similarly, strain tensor $\varepsilon_{ij}$ can be decomposed into spherical strain tensor $\varepsilon_{m}$ and deviatoric strain tensor $e_{ij}$ [20,21]


$$\begin{array}{c} \left\{ \begin{array}{c} \sigma_{ij}=S_{ij}+\delta_{ij}\sigma_{m}\\ \varepsilon_{ij}=e_{ij}+\delta_{ij}\varepsilon_{m} \end{array}\right. \end{array}$$ (36)

In a three-dimensional state, the strain and stress satisfy the following conditions.


$$\begin{array}{c} \left\{ \begin{array}{c} e_{ij}=\frac{1}{2G}S_{ij}\\ \varepsilon_{kk}=\frac{1}{3K}\sigma_{kk} \end{array}\right. \end{array}$$ (37)

where $S_{ij}$ is stress tensor, $e_{ij}$ is the strain partial tensor, $\varepsilon_{kk}$ is the first invariant of the strain tensor, $\sigma_{kk}$ is the first invariant of the stress tensor, *G* is the shear modulus, *K* is the bulk modulus.

According to the analogy method and Eqs. (12), (36), (37), we get


$$\begin{array}{c} \varepsilon_{11}^{1}=\frac{\sigma_{1}-\sigma_{3}}{3G_{1}}+\frac{\sigma_{1}+2\sigma_{3}}{9K_{1}} \end{array}$$ (38)

where *G*_1_ is the instantaneous shear modulus, *K*_1_ is the instantaneous bulk modulus, $\varepsilon_{11}^{1}$ is the instantaneous strain, $\sigma_{1}$ is the axial stress, $\sigma_{3}$ is the confining pressure.

According to the analogy method and Eqs. (22), (36), (37), we get


$$\begin{array}{c} \varepsilon_{11}^{2}=\frac{\sigma_{1}-\sigma_{3}}{3G_{2}}\left\{1-\exp\left\{-\frac{\beta_{2}t^{\beta_{2}}}{2H_{2}p_{2}}{\left[-Z_{2T}{\left(\frac{t}{t_{c}}\right)}^{p_{2}}\right]}^{-\frac{\beta_{2}}{p_{2}}}\Gamma \left[\frac{\beta_{2}}{p_{2}},-Z_{2T}{\left(\frac{t}{t_{c}}\right)}^{p_{2}}\right]\right\}\right\} \end{array}$$ (39)

where $\varepsilon_{11}^{2}$ is the viscoelastic strain, *G*_2_ is the viscoelastic shear modulus, *H*_2_ is the viscoelastic viscosity coefficient.

Viscoplastic strain cannot be directly transformed by analogy. Viscoplastic strain requires yield function derivation. The yield function is [22]


$$\begin{array}{c} F=\sqrt{J_{2}}-\frac{\sigma_{s}}{\sqrt{3}}=\frac{\sigma_{1}-\sigma_{3}-\sigma_{s}}{\sqrt{3}} \end{array}$$ (40)

where *J*_2_ is the second invariant of the stress tensor, *F* is the yield function.

Viscoplastic strain is

When $\sigma \leq \sigma_{s}$,


$$\begin{array}{c} \varepsilon_{11}^{3}=0 \end{array}$$ (41)

When $\sigma >\sigma_{s}$,


$$\begin{array}{c} \varepsilon_{11}^{3}=-\frac{F\beta_{3}}{2H_{3}}\frac{\partial F}{\partial \sigma_{11}}{\left\{\left[Z_{3\sigma }\left(\sigma_{1}-\sigma_{3}-\sigma_{s}\right)-\frac{Z_{3T}}{t_{c}}\right]t\right\}}^{-\beta_{3}}\Gamma \left\{\beta_{3},\left[Z_{3\sigma }\left(\sigma_{1}-\sigma_{3}-\sigma_{s}\right)-\frac{Z_{3T}}{t_{c}}\right]t\right\} \end{array}$$ (42)

where $\varepsilon_{11}^{3}$ is the viscoplastic strain, *H*_3_ is the viscoplastic fractional viscosity coefficient damaged.

By substituting Eq. (40) into Eq. (42), we obtain


$$\begin{array}{c} \varepsilon_{11}^{3}=-\frac{\left(\sigma_{1}-\sigma_{3}-\sigma_{s}\right)\beta_{3}}{2\sqrt{3}H_{3}}{\left\{\left[\frac{Z_{3\sigma }\left(\sigma_{1}-\sigma_{3}-\sigma_{s}\right)}{\sqrt{3}}-\frac{Z_{3T}}{t_{c}}\right]t\right\}}^{-\beta_{3}}\Gamma \left\{\beta_{3},\left[\frac{Z_{3\sigma }\left(\sigma_{1}-\sigma_{3}-\sigma_{s}\right)}{\sqrt{3}}-\frac{Z_{3T}}{t_{c}}\right]t\right\} \end{array}$$ (43)

The total strain of rock $\varepsilon_{11}$ is [23,24]


$$\begin{array}{c} \varepsilon_{11}=\varepsilon_{11}^{1}+\varepsilon_{11}^{2}+\varepsilon_{11}^{3} \end{array}$$ (44)

By substituting Eqs. (38), (39), (41) and (43) into Eq. (44), we obtain

When $\sigma \leq \sigma_{s}$,


$$\begin{array}{cc} \varepsilon_{11}= & \frac{\sigma_{1}-\sigma_{3}}{3G_{1}}+\frac{\sigma_{1}+2\sigma_{3}}{9K_{1}}\\ & +\frac{\sigma_{1}-\sigma_{3}}{3G_{2}}\left\{1-\exp\left\{-\frac{\beta_{2}t^{\beta_{2}}}{2H_{2}p_{2}}{\left[-Z_{2T}{\left(\frac{t}{t_{c}}\right)}^{p_{2}}\right]}^{-\frac{\beta_{2}}{p_{2}}}\Gamma \left[\frac{\beta_{2}}{p_{2}},-Z_{2T}{\left(\frac{t}{t_{c}}\right)}^{p_{2}}\right]\right\}\right\} \end{array}$$ (45)

When $\sigma >\sigma_{s}$,


$$\begin{array}{cc} \varepsilon_{11}= & \frac{\sigma_{1}-\sigma_{3}}{3G_{1}}+\frac{\sigma_{1}+2\sigma_{3}}{9K_{1}}\\ & +\frac{\sigma_{1}-\sigma_{3}}{3G_{2}}\left\{1-\exp\left\{-\frac{\beta_{2}t^{\beta_{2}}}{2H_{2}p_{2}}{\left[-Z_{2T}{\left(\frac{t}{t_{c}}\right)}^{p_{2}}\right]}^{-\frac{\beta_{2}}{p_{2}}}\Gamma \left[\frac{\beta_{2}}{p_{2}},-Z_{2T}{\left(\frac{t}{t_{c}}\right)}^{p_{2}}\right]\right\}\right\}\\ & -\frac{\left(\sigma_{1}-\sigma_{3}-\sigma_{s}\right)\beta_{3}}{2\sqrt{3}H_{3}}{\left\{\left[\frac{Z_{3\sigma }\left(\sigma_{1}-\sigma_{3}-\sigma_{s}\right)}{\sqrt{3}}-\frac{Z_{3T}}{t_{c}}\right]t\right\}}^{-\beta_{3}}\Gamma \left\{\beta_{3},\left[\frac{Z_{3\sigma }\left(\sigma_{1}-\sigma_{3}-\sigma_{s}\right)}{\sqrt{3}}-\frac{Z_{3T}}{t_{c}}\right]t\right\} \end{array}$$ (46)

Eqs. (45) and (46) are the triaxial creep damage model of rock considering the influence of fractional order and temperature.

## 4 Creep test of rock under different temperatures

### 4.1 Mechanical properties test

The rock samples used in this study are collected from an underground roadway located in Dandong City, Liaoning Province. The tunnel face is buried at a depth of approximately 600 m, where the in-situ stress is measured to range from 9.15 MPa to 11.36 MPa. Considering the actual stress environment and to facilitate the subsequent triaxial creep test loading scheme, a confining pressure of 10 MPa is adopted in the test, which is within the range of the measured in-situ stress and can reasonably reflect the real stress state of the surrounding rock. The basic mechanical parameters of this type of rock mass are approximately as follows. The density is about 2500–2700 kg m^3^. The Poisson’s ratio is 0.22–0.30. The cohesion is 8–15 MPa, and the internal friction angle is 35°–45°. The test is carried out using a fully automatic triaxial testing apparatus(Taw-2000). The axial pressure of this device can reach up to 2000 kN, with a maximum confining pressure of 60 MPa, which can fully meet the requirements of triaxial creep and strength tests under in-situ stress conditions.

Before carrying out the rock creep test, it is necessary to carry out the rock mechanical properties test under the corresponding conditions. The stress-strain curves of rock under different temperatures clearly reveal a significant temperature-induced mechanical deterioration effect. As the temperature increases from 25 °C to 100 °C, both the peak stress and residual strength of the rock gradually decrease, dropping from approximately 92 MPa and 68 MPa at 25 °C to around 65 MPa and 33 MPa at 100 °C, respectively. Meanwhile, the strain corresponding to the peak stress shifts to a lower range, indicating that the rock exhibits a transition from brittle to more ductile behavior under elevated temperatures. The slopes of the initial linear elastic segments also decrease with increasing temperature, reflecting a reduction in the elastic modulus and stiffness of the rock. These trends confirm that high temperatures exacerbate thermal damage, promoting the initiation and propagation of microcracks, weakening intergranular bonds, and consequently degrading the bearing capacity and deformation resistance of the rock, which provides direct experimental evidence for the temperature-dependent mechanical degradation and creep damage evolution mechanism considered in the proposed model.

### 4.2 Temperature-stress coupling creep characteristic test

The specific steps of the temperature-stress coupling rock triaxial creep test are as follows [25–27].

1. In accordance with the ISRM standards, the rock samples collected from the roadway in Dandong were processed into cylindrical specimens with a diameter of 50 mm and a height of 100 mm. After measuring their size, mass, natural water content and initial wave velocity, the specimens with obvious cracks and defects were eliminated.
2. The specimens were divided into four temperature groups (25 °C, 50 °C, 75 °C and 100 °C). Multiple stress levels were set for each group, and at least 3 parallel specimens were prepared in each group to ensure the reliability of test results.
3. A fully automatic triaxial creep testing machine (TAW-2000) was adopted. The equipment, including the axial pressure, confining pressure, temperature control system and displacement sensor, was inspected and debugged in advance. The data acquisition system was connected to record stress, strain, temperature and time synchronously.
4. The specimen was sleeved with a heat-shrinkable tube and installed in the pressure chamber, with insulating gaskets placed at both ends. Axial and radial extensometers were attached to the specimen surface to ensure close fit without affecting subsequent loading and heating.
5. The confining pressure was applied to 10 MPa at a constant rate of 0.05 MPa/s and kept stable. A small axial preload (1–2 MPa) was applied to eliminate the gap between the indenter and the specimen end face, and the extensometers were zeroed for initial state calibration.
6. The temperature control system was started to heat the specimen to the target temperature at a rate of 1 °C min^-1^ to avoid thermal shock cracking. After reaching the target temperature, constant temperature insulation was maintained for at least 2 hours to ensure uniform internal temperature distribution of the specimen.
7. Axial stress was applied step by step at a rate of 0.05–0.1 MPa/s. After each level of stress was stably applied, creep data was collected continuously. If the axial strain rate was less than $1\times 10^{-6}/h$ for 48 consecutive hours, the next level of stress was applied; if the specimen entered the accelerated creep stage with a continuously increasing strain rate, loading was continued until specimen failure. The confining pressure and temperature were kept constant throughout the test.
8. After the specimen failed or the predetermined test duration was reached, stress loading was stopped first, and then the confining pressure was slowly unloaded at a rate of 0.05 MPa/s. The temperature system was turned off to let the specimen cool to room temperature. The specimen was taken out, its failure morphology was observed and recorded, and the pressure chamber and equipment were cleaned.
9. The collected stress, strain, temperature and time data were sorted into time series. Creep curves under different temperatures and stress levels were drawn, and characteristic parameters of the three creep stages were extracted for subsequent model verification and analysis.

It can be seen that temperature and stress are the core two factors that regulate the creep characteristics of rock. As the temperature gradually increases from 25 °C to 100 °C, the long-term strength of rock deteriorates significantly, and the initial stress of accelerated creep continues to decrease from 85 MPa to 60 MPa. High temperature greatly accelerates the initiation, propagation and penetration of microcracks by triggering thermal damage effects such as mineral phase transformation, grain boundary cracking and pore expansion, so that the critical threshold of rock from stable creep to accelerated failure is advanced. Under the same temperature conditions, the creep behavior shows a typical three-stage characteristic of primary-steady-stable-acceleration. At low stress levels, it only shows initial deceleration creep and subsequent steady-state creep, and the strain rate gradually stabilizes and maintains a constant without obvious failure trend. When the stress exceeds the critical value at the corresponding temperature, the rock will quickly enter the accelerated creep stage, the strain rate rises sharply, and finally approaches failure. The coupling effect of high temperature and high stress further magnifies the deformation and damage degree of rock. The working condition of 60 MPa at 100 °C enters severe accelerated creep only within more than ten hours, which fully reflects the synergistic evolution of thermal damage and stress creep damage. The deterioration of rock mechanical properties provides key experimental basis and theoretical support for long-term stability evaluation, structural design and rheological disaster prevention and control of high-temperature rock mass engineering such as deep mineral mining, geothermal development and nuclear waste disposal.

According to the literature [28–30], the stress corresponding to the stress divergence point is the long-term strength of the rock. The long-term strength of the rock at 25 °C is 75 MPa.

## 5 Parameter identification and model verification

The model parameters are identified by the least squares method, which aims to minimize the sum of squared differences between the experimental creep strains and the model-predicted strains. By constructing an objective function with the residual error as the core, an iterative optimization algorithm is used to continuously adjust the parameters such as elastic modulus, fractional viscosity coefficient, fractional order, and damage parameters. When the sum of squared residuals reaches the minimum, the corresponding parameter combination is regarded as the optimal solution, so that the theoretical curve is in the best agreement with the test data [30–33].

The proposed fractional-order creep damage model considering temperature effects can accurately characterize the full-stage creep behavior of rock under coupled temperature and stress conditions. The model-predicted curves show excellent agreement with the experimental creep curves, fully reproducing the three-stage creep characteristics (decelerated, steady-state, and accelerated creep) under various working conditions (25 °C, 50 °C, 75 °C, and 100 °C). The trends, critical inflection points, and final strain magnitudes of the model predictions are highly consistent with the test data. Quantitatively, the correlation coefficients (*R*^2^) between the model and experimental curves all exceed 0.95, demonstrating that the model effectively captures the synergistic effects of thermal degradation, fractional-order rheology, and damage evolution on the long-term mechanical behavior of rock. This confirms the model’s high descriptive accuracy and reliability, providing robust theoretical support for the long-term stability analysis of deep high-temperature rock engineering.

The root mean square error (RMSE) values of the proposed model under different stress levels range from 0.0089% to 0.0214%, which are consistently low across all conditions. Combined with the correlation coefficients *R*^2^ > 0.95 for all cases, the results further demonstrate that the model predictions are in excellent agreement with the experimental data, confirming its high accuracy and reliability in describing the full-stage creep behavior of rock under temperature-stress coupling conditions.

To verify the superiority and reliability of the proposed fractional-order creep damage model, a comparative analysis is conducted with the classical Nishihara model and a previously reported rheological model [28,27]. The comparison reveals that both the Nishihara model and the existing model can roughly describe the decelerated and steady-state creep stages, but they fail to accurately capture the accelerated creep stage induced by thermal damage and stress-induced crack propagation, leading to noticeable deviations between the predicted curves and the test data, especially in the late deformation period. In contrast, the proposed model, which incorporates temperature-dependent fractional-order rheology and coupled thermal-stress damage evolution, not only precisely reproduces the entire three-stage creep process but also aligns closely with the experimental inflection points and strain magnitudes. Quantitative evaluation further confirms that the correlation coefficients (*R*^2^) of the proposed model exceed 0.95 for all working conditions, which are significantly higher than those of the Nishihara model and the existing model, demonstrating its superior descriptive accuracy and broader applicability in characterizing the long-term rheological behavior of rocks under high-temperature environments.

Parameter sensitivity analysis demonstrates that the proposed fractional-order creep model can accurately capture the regulatory mechanisms of key physical parameters on creep behavior. Specifically, an increase in the time damage exponent *p*_2_ significantly elevates the creep strain level and accelerates the transition to the accelerated creep stage in the later phase, reflecting its role in controlling the nonlinear accumulation rate of temperature-time coupled damage. Larger values of the fractional-order exponents $\beta_{2}$ (viscoelastic phase) and $\beta_{3}$ (viscoplastic phase) respectively smooth the viscoelastic creep curve and intensify the upward inflection of the viscoplastic phase, illustrating the regulatory effects of fractional-order parameters on the elastic-viscous transition characteristics and nonlinear viscoplastic flow of the material. Increases in the temperature damage coefficients *Z*_2 *T*_ and *Z*_3 *T*_ both suppress creep deformation remarkably and reduce the overall strain level, embodying the thermal hardening/damage saturation effects induced by high temperatures. In contrast, a larger stress damage coefficient $Z_{3\sigma }$ drastically increases the accelerated creep rate and accelerates material failure, highlighting the strong driving effect of deviatoric stress on damage evolution. The above parameter response laws clearly verify the rationality and effectiveness of the proposed model in characterizing rock creep behavior under the coupling of temperature, stress, time, and fractional-order characteristics.

## 6 Conclusions

The main innovations of this paper are as follows. Different from the existing models that simply modify the viscosity coefficient with temperature or treat thermal damage as a static variable, this paper constructs a time-varying thermal damage evolution equation that depends on both the temperature level and the high temperature action time. Combined with the stress creep damage equation considering the Arrhenius temperature effect, the synergistic coupling of thermal-stress double time-varying damage is realized. The traditional Nishihara model is optimized by introducing a temperature-related fractional Kelvin element. The explicit analytical solution of the model is obtained through strict mathematical derivation, and the uniaxial model is further extended to a triaxial creep damage model suitable for complex engineering stress conditions. It not only makes up for the shortcomings of traditional models in describing accelerated creep, but also accurately characterizes the memory and genetic rheological properties of rock with fewer parameters, bridging the gap between laboratory test research and engineering field application.

To address the limitations of existing rock creep models, this study constructs a time-varying thermal damage evolution equation dependent on both temperature and duration, and realizes the synergistic coupling of thermal-stress dual time-varying damage by combining it with a stress creep damage equation considering the Arrhenius temperature effect. Based on fractional calculus and continuum damage mechanics, an improved fractional-order creep damage model is established by optimizing the Nishihara model and introducing temperature-affected fractional viscosity coefficients, which can accurately characterize the full three-stage creep behavior of rock and effectively capture the nonlinear rheological characteristics induced by temperature. Verification via rock creep tests under multiple temperature and stress levels shows that the proposed model has high fitting accuracy and obvious superiority over the classical Nishihara model and existing similar models, especially in capturing the accelerated creep inflection point. Furthermore, the derived triaxial creep damage model expands the model’s application to complex engineering stress conditions, providing important theoretical support and technical reference for the long-term stability evaluation and rheological disaster prevention of deep high-temperature rock engineering.
